# Caregiver burden and parenting stress among left-behind elderly individuals in rural China: a cross-sectional study

**DOI:** 10.1186/s12889-021-10892-9

**Published:** 2021-05-01

**Authors:** Mengjuan Zhao, Ziqiang Zhu, Chenchen Kong, Chunshan Zhao

**Affiliations:** Department of Community Nursing, School of Nursing, Beihua University, Jilin, China

**Keywords:** Caregiver burden, Parenting stress, Left-behind elderly

## Abstract

**Background:**

One public health problem that cannot be ignored is the mental health of left-behind elderly individuals in rural areas. However, the burden of care and parenting stress among left-behind elderly individuals has never been analyzed. The purpose of this study was to explore the level of caregiver burden and parenting stress and their relationship among left-behind elderly individuals.

**Methods:**

A total of 261 left-behind elderly people responded to the study. The 22-item Zarit Burden Interview and the 36-item Parenting Stress Index-Short Form were used.

**Results:**

We sent out 300 questionnaires in total. The effective rate was 87% (*n* = 261). Among the left-behind elderly individuals, most respondents were female (*n* = 171; 65.5%). The results showed that older age (OR:3.04; 95%CI: 1.307–7.048), an annual income of ¥5000–¥9900 (OR:3.25; 95%CI: 1.192–8.852) and higher parenting stress (OR:1.17; 95%CI: 1.103–1.242) were the risk factors related to higher caregiver burden in the left-behind elderly individuals. The influencing factor for lower caregiver burden in the left-behind elderly was gender (being male) (OR:0.08; 95%CI:0.036–0.178). Age (r = − 0.789; *P* < 0.001) and gender (r = 0.325; *P* < 0.001) were significant positively correlated with parenting stress, and annual income (r = − 0.717; *P* < 0.001) was negatively correlated with parenting stress.

**Conclusion:**

Parenting stress is a risk factor affecting caregiver burden of left-behind elderly individuals. Healthcare professionals should pay close attention to the caregiver burden and parenting stress of left-behind elderly individuals, especially those who are older, female and have lower income.

## Background

Intensified rural–urban population migration coupled with globalization has brought about tremendous changes in rural family lifestyle, function and structure in a number of developing countries. China is currently the largest nation of immigrants on a global scale. In 2000, the floating population totaled 79 million [1]. By 2017, this figure had increased to approximately 250 million. Furthermore, China is a country with the oldest population. More than 250 million people were above 60 years old in 2018 [2]. By 2030, this figure will be approximately 300 million and will reach approximately 400 million by 2050 [3]. Immigration often leads to a large number of left-behind elderly people in rural areas. The population of rural left-behind elderly individuals is a special vulnerable group that refers to old parents living alone after their children leave the countryside to work or do business for more than 6 months [4]. In rural areas of China, most left-behind elderly individuals are influenced by the Chinese value of filial piety, and will take the initiative to care for their grandchildren. However, as time progresses, due to influences from economic resources and contemporary individualism, the family status of rural grandparents have started to shift [5]. For the elderly, the act of raising grandchildren is considered a reciprocal form of intergenerational exchange rather than an emotional reward [6]. Left-behind elderly individuals often take care of their grandchildren and have shown to have a heavy burden and considerable stress.

In China, the level of caregiver burden may be greater than that in developed countries because of the absence of a perfect community-based system of care [7]. Caregiver burden is a psychological term that refers to caregiving having had a negative impact on emotional, social, financial, physical, and spiritual functioning [8, 9]. Caregiver burden is multidimensional and complex. Rural left-behind elderly individuals not only have to take care of themselves but also their grandchildren. Caregivers often report anxiety, depression, and fear [10–12]. This burden is often multidimensional, as it includes both subjective and objective burden. Subjective burden could result from stress, and the two types of burdens influence each other.

Parenting stress has been defined as the experience of distress or discomfort that results from demands associated with the role of parenting [13]. The experience of parenting children can be stressful, especially left-behind children. A number of studies have documented that parenting stress has more physical and psychological health effects on themselves and their children [14–16]. Along with parenting stress, caregiver burden appears to be a strong contributor to symptoms of depression and anxiety among left-behind elderly individuals as well as an increased risk of disease. Research on caregiver burden and parenting strain among rural left-behind elderly individuals has vital significance.

To date, studies published on the burden and strain of Chinese rural left-behind elderly individuals are not available. We hypothesized that the levels of caregiver burden and parenting stress were high among these individuals.

## Methods

### Participants

From October 2017 to March 2018, a cross-sectional investigation and descriptive study were conducted on rural left-behind elderly people in Jilin, China. Based on the geographic distribution of Jilin, the whole city was divided into 5 counties: one county was randomly selected from the city, three towns were randomly selected from the county, and two villages were randomly selected from each town, a total of six villages were selected. A total of 300 individuals were investigated in the study. The inclusion criteria were as follows: (1) ≥60 years old; (2) adult children not present for more than half a year; (3) clear awareness and communication skills; and (4) grandchildren for more than 1 year. The exclusion criteria were as follows: (1) not rural left-behind elderly individuals; (2) health status or communication skills can affect results.

### Measures

#### Caregiver burden scale

The burden of caregivers was evaluated with the Zarit Caregiver Burden Inventory (ZBI), which uses 22 items to assess the burden level of caregivers in health, mental state, economic and social life [17]. Each item was answered using a five-point Likert scale (0 “never” to 4 “nearly always”). The total score ranges from 0 to 88 points. The burden score was segmented into 0–40 (mild burden), 41–60 (moderate burden), and 61–88 (severe burden). The Chinese version of the ZBI (C-ZBI) was used for this study, and it has been shown to have good validity and internal consistency reliability (Cronbach’s α = 0.87) [18].

#### Parenting stress index-short form

The Chinese version of the PSI-SF, which is taken from Abidin’s version [19], was utilized to measure the stress in the parenting role [20]. It is a 36-item tool designed for the assessment of parenting stress in three factors: parental distress, parent–child dysfunctional interaction, and difficult child. Similar to previous studies [21], we changed “parents” to “grandparents” and “child” to “grandchildren” for some items. Each item was scored on a five-point Likert scale, from 1 (strongly disagree) to 5 (strongly agree), and higher scores represent a higher perceived level of parenting stress. In the current study, the Chinese version of the PSI-SF has been used in a large number of populations and has adequate evidence to support its reliability and validity. Cronbach’s α coefficient is excellent (0.90).

#### Data collection

The rural left-behind elderly individuals were interviewed face to face by a well-trained investigator in their home. Printed questionnaires were sent out and were collected in person. In the course of the investigation, unified instructions were adopted, and the subjects were required to independently finish the questionnaires. If the subject was unable to complete the questionnaire, the investigator assisted in completing the questionnaire. A total of 39 invalid questionnaires were removed from the 300 completed questionnaires. Finally, 261 valid responses were included for data analysis, with an effective rate of 87.0%.

#### Ethical aspects

Participants were told that the study was voluntary and that they could withdraw from the study at any time. All questionnaires were completed anonymously. The Ethics Committee approved this study of Beihua University.

#### Data analysis

SPSS 20.0 was used for data analysis. Frequencies and percentages were used to analyze the social demographic characteristics of left-behind elderly individuals and children. Multiple logistic regression analysis and Pearson correlation analysis were used to determine the factors affecting the caregiver burden and parenting stress of the left-behind elderly. Multiple logistic regression analysis was used to determine the parenting stress factors affecting the level of burden among left-behind elderly individuals.

A significance level of *P* < 0.05 was considered significant.

## Result

### Participants

We sent out a total of 300 questionnaires. The effective rate was 87% (*n* = 261). A total of 39 questionnaires were excluded due to non-response bias, which could throw off the analysis results. Among the grandchildren, when compared to non-respondents, the respondents were primarily girls (*n* = 156; 59.8%). Among the rural left-behind elderly, when compared to non-respondents, most respondents were female (*n* = 171; 65.5%) and married (*n* = 234; 89.7%) (Table 1).

**Table 1:** Distribution of non-respondents and respondents according to characteristics

Variable	Non-respondents(*n* = 39)		Respondents(*n* = 261)		*p*-value^a^
Grandchildren	n	%	n	%	
Age (years)					
1–3	7	17.9	30	11.5	0.010
4–6	13	33.3	75	28.7	
≥ 7	19	48.7	156	59.8	
Gender					
Girls	16	41.0	156	59.8	0.027
Boys	23	59.0	105	40.2	
The rural left-behind elderly					
Age (years)					
60–69	21	53.8	152	58.2	0.872
70–79	14	35.9	84	32.2	
≥ 80	4	10.3	25	9.6	
Gender					
Female	17	43.6	171	65.5	0.008
Male	22	56.4	90	34.5	
Marital status					
Married	27	69.2	234	89.7	< 0.001
Widowed	9	23.1	24	9.2	
Divorced/separated	3	7.7	3	1.1	
Annual income					
Less than ¥5000(<$710)	5	12.8	30	11.5	0.748
¥5000–¥9900($710–$1410)	11	28.2	73	28.0	
¥10,000–¥14,900($1420–$2110)	8	20.5	74	28.4	
More than ¥15,000(≥$2130)	15	38.5	84	32.2	

^a^Chi-square test

### Association between caregiver burden and the characteristics of rural left-behind elderly individuals

The average scores of each item in the C-ZBI are shown in Table 2. The following items had the highest average scores: there was not enough free time for yourself (item 2), loss of control of life (item 17), feeling stressed (item 3), grandchildren depending on you (item 8) and privacy (item 11). The following items had the lowest average scores: state of health (item 10), grandchildren’s friends come to visit (item 13) and strain (item 9).

**Table 2:** Item Scores of the C-ZBI (*n* = 261)

C-ZBI item (n = 261)	Mean	SD	Min	Max
Factor 1 The caregivers’ feeling of over sacrifice				
16. Do you feel that you will be not able to take care of your relative much longer?	2.03	0.87	0	4
18. Do you wish you could just leave the care of your relative to someone else?	1.97	1.43	0	4
15. Do you feel that you don’t have enough money to care for your relative?	1.36	1.05	0	4
2. Do you feel that because of the time you spend with your relative that you don’t have enough time for yourself?	2.87	0.91	1	4
22. Overall, how burdened do you feel in caring for your relative?	1.75	1.29	0	4
10. Do you feel your health has suffered because of your involvement with your relative?	1.02	1.01	0	3
17. Do you feel that you have lost control of your life since your relative’s illness?	2.85	0.82	0	4
3. Do you feel stressed between caring for your relative and trying to meet other responsibilities for your family or work?	2.69	0.94	1	4
Factor 2 The patients’ dependence on the caregiver				
8. Do you feel your relative is dependent on you?	2.87	0.87	0	4
14. Do you feel that your relative seems to expect you to take care of him/her, as if you were the only one he/she could depend on?	2.54	0.89	0	4
11. Do you feel that you don’t have as much privacy as you would like because of your relative?	2.62	0.92	1	4
12. Do you feel that your social life has suffered because you are caring for your relative?	2.40	1.02	0	4
Factor 3 The negative emotion due to caring				
4. Do you feel embarrassed over your relative’s behavior?	2.01	0.92	0	4
13. Do you feel uncomfortable about having friends over because of your relative?	0.79	0.97	0	4
6. Do you feel that your relative currently affect your relationship with other family members or friends in a negative way?	1.23	0.80	0	3
5. Do you feel angry when you are around your relative?	1.36	0.96	0	3
9. Do you feel strained when you are around your relative?	0.76	0.84	0	4
Factor 4 Caregiver’s feeling of inadequacy				
20. Do you feel you should be doing more for you relative?	1.68	0.69	0	3
21. Do you feel you could do a better job in caring for your relative?	1.70	0.65	1	3
Factor 5 Uncertainty about patient’s future				
7. Are you afraid of what the future holds for your relative?	1.86	0.97	0	4
19. Do you feel uncertain about what to do about your relative?	1.99	0.80	0	4
1. Do you feel that your relative asks for more help than he/she needs?	2.14	1.07	0	4
Zarit total	42.47	11.51		

Multiple logistic regression analysis was conducted by taking caregiver burden level as the dependent variable, and age, gender, marital status, annual income and C-PSF score as the independent variables. The results showed that the risk factors causing higher caregiver burden in the left-behind elderly were increasing age, an annual income in the ¥5000–¥9900 range, and higher parenting stress. The influencing factor for lower caregiver burden in the left-behind elderly was gender (being male) (Table 3).

**Table 3:** Multiple logistic regression analysis of different variables in relation to C-ZBI scores (n = 261)

Variable	***β***	***OR***	***95%CI***	***p***
Age	1.110	3.04	1.307–7.048	0.010
Gender (male)	−2.527	0.08	0.036–0.178	< 0.001
Marital status (widowed)	0.828	2.29	0.352–1.247	0.199
Marital status (divorced/separated)	0.044	1.05	0.058–1.853	0.978
Annual income ¥5000–¥9900($710–$1410)	1.178	3.25	1.192–8.852	0.021
Annual income ¥10,000–¥14,900($1420–$2110)	0.719	2.05	0.645–6.530	0.223
Annual income More than ¥15,000(≥$2130)	−0.398	0.67	0.379–1.191	0.173
C-PSF scores	0.158	1.17	1.103–1.242	< 0.001

### Association between parenting stress and the characteristics the rural left-behind elderly individuals

Table 4 shows the associations between each variable and the C-PSF scores. Age and gender were significant positively correlated with parenting stress. Annual income was negatively correlated with parenting stress.

**Table 4:** Correlation analysis of different variables in relation to C-PSF scores (*n* = 261)

Variable	Pearson correlation	
	r	*p*
Age (years)	−0.789	< 0.001
Gender	0.325	< 0.001
Marital status	− 0.021	0.738
Annual income	−0.717	< 0.001

## Discussion

Several studies have shown that the health risks of left-behind elderly people may be related to the migration of adult children [22, 23]. Adult children often leave their children in the care of left-behind parents when they migrate. This alleviates the loneliness of the elderly individuals to some extent but also increases their burden of care and the pressures related to parenting children. However, there are no public studies on the burden of care and parenting pressure of left-behind elderly people. Assessing the burden and strain in left-behind elderly people carries significance for establishing standardized rural community health services in the future. In this study, we demonstrated the level and relationship between the burden of care and parenting pressure among left-behind elderly people.

The level of caregiver burden and parenting stress is related to the age, gender and annual income of left-behind elderly people. There was a positive correlation between age and burden, whereas there was a negative correlation between age and stress. On the one hand, the deterioration of body functions and the increase in chronic diseases lead to increased psychological and physical burden among elderly people. While fulfilling their guardianship responsibilities, they also have to take care of their own health status and bear a heavy burden of labor. On the other hand, older adults are getting better at dealing with negative emotions and coping strategies with increasing age [24]. In addition, having more parenting experience may be the reason for their lower parenting stress. Females are positively correlated with burden and parenting stress, which is consistent with most studies [25–27]. In traditional Chinese culture, females assume the role of caregivers and are responsible for family affairs [28]. As a result, they are less likely to have formal or informal social support than male caregivers [29]. Furthermore, males are more likely than females to deal with burdens and stress, such as focusing on tasks and blocking emotions [30]. Families with higher annual incomes will bear less burden and parenting stress. Higher incomes can improve their living conditions and reduce the difficulties they face in raising grandchildren.

Parenting stress is a risk factor affecting caregiver burden of left-behind elderly individuals. From a social point of view, left-behind elderly people need more social support, pension security and health resources to ease the difficulties in raising grandchildren. From a personal point of view, they need more time to do what they want to do. Previous studies have found that parental migration has many negative effects on left-behind children, such as social anxiety, depression, learning counseling and inadequate supervision [31, 32]. These problems bring obstacles to the work of raising grandchildren. Increasing psychological counseling and effective intervention for left-behind elderly individuals and children can effectively reduce the burden and parenting pressure experienced by caregivers.

## Conclusion

Parenting stress experienced by left-behind elderly individuals is a risk factor affecting the burden on their caregivers. Consequently, healthcare professionals should pay close attention to the caregiver burden and parenting stress of left-behind elderly individuals, especially those who are older, female and have lower income. To reduce their psychological burden and pressure, elderly individuals with high burden and pressure levels should receive psychological counseling to assist them to understand current attitudes about intergenerational rearing. From a practical point of view, the present study can provide academic support to government departments when they formulate relevant policies to safeguard the interests of left-behind elderly individuals.

## Data Availability

The datasets used and analyzed during the current study are available from the corresponding author on reasonable request.

## Abbreviations

ZBI: The Zarit Caregiver Burden Inventory
C-ZBI: The Chinese version of the ZBI
PSI-SF: Parenting Stress Index-Short Form
SPSS: Statistical Product and Service Solutions
C-PSF: the Chinese version of the Parenting Stress Index-Short Form
